# Mini-puberty testosterone and infant autistic traits

**DOI:** 10.3389/fendo.2023.1126023

**Published:** 2023-04-05

**Authors:** Alex Tsompanidis, Sarah Hampton, Ezra Aydin, Carrie Allison, Rosemary Holt, Simon Baron-Cohen

**Affiliations:** ^1^ Autism Research Centre, Department of Psychiatry, University of Cambridge, Cambridge, United Kingdom; ^2^ York Trials Unit, University of York, York, United Kingdom; ^3^ Vagelos College of Physicians and Surgeons, Columbia University, New York, NY, United States

**Keywords:** autism, mini-puberty, testosterone, infant, autistic traits, longitudinal

## Abstract

**Background:**

Levels of steroid hormones in the first three months of life, a period referred to as ‘mini-puberty’, are one of the earliest physiological differences between typical males and females postnatally. Autistic traits also show consistent typical sex differences in later infancy, after the 18^th^ month of life. Previous studies have shown *prenatal* testosterone is associated with later levels of autistic traits. Studies testing if postnatal testosterone levels are associated with autistic traits have reported null results. No studies to date have investigated mini-puberty longitudinally or tested for interactions with baseline sex differences or familial likelihood of autism.

**Methods:**

The ‘Cambridge Human Imaging and Longitudinal Development Study’ (CHILD) is a prospective enriched cohort study in Cambridge, UK. It includes physiological measurements in early infancy, as well as neurodevelopmental follow-ups over the first two years of life. A subset of the cohort also includes children with a family history of autism (a diagnosed parent or sibling). Salivary testosterone levels were assessed at two time-points, just after the 2^nd^ and 6^th^ month of life. Autistic traits were measured using the Quantitative Checklist of Autism in Toddlers (Q-CHAT) when the children were 18 months of age.

**Results:**

Salivary testosterone levels were significantly higher during ‘mini-puberty’ in the 2^nd^ and 3^rd^ month of life, compared to after the 6^th^ month of life, in both males and females. There was no significant sex difference at either time-point. Log-transformed testosterone levels were not associated with autistic traits (Q-CHAT). There was no interaction effect with infant sex, autism family history or baseline testosterone levels after mini-puberty (at >6 months of age).

**Conclusion:**

Both male and female infants have elevated levels of salivary testosterone during mini-puberty but in this relatively small sample this was not associated with their later autistic traits at 18 months or their family history of autism. This suggests that *prenatal* rather than postnatal testosterone levels are more relevant for understanding the causes of autism. Future studies should test these relationships in larger samples.

## Introduction

Autism is a neurodevelopmental condition that combines difficulties in social interaction and communication, alongside unusually restricted interests and repetitive behaviours. Based on twin-heritability estimations, gene sequencing and genome-wide-association studies that capture common variance, there is a consensus that most of the liability in autism (diagnosis or traits) can be attributed to genetics (1, 2).

A diagnosis of (non-syndromic) autism remains behavioural and is only possible after 18-months of age, when specific neurodevelopmental behaviours emerge in toddlerhood, specifically in relation to social attention, language use and play. The Q-CHAT (or Quantitative Checklist for Autism in Toddlers) is a psychometric measure, rated by a parent or care-giver, developed to capture these traits in an additive way, and indexing a continuous spectrum (3). Several studies have confirmed its validity in predicting a later autism diagnosis in children *via* gold-standard neurodevelopmental assessments by specialists (i.e., based DSM-IV/5 or ICD-10 criteria). Validation studies for the Q-CHAT have been conducted in different populations of different ethnicities, with consistent results (4–6).

Interestingly, a sex difference in Q-CHAT scores has been noted in many of these studies, with males scoring higher in terms of autistic traits at 2 years of age. This is consistent with early sex differences in several socio-cognitive milestones, such as language development (7, 8), as well as in autistic traits in childhood and adulthood, which have been consistently found to be higher in males (9). This could be attributed to potential biases in terms of gendered behaviours that are not well characterised in young females (10). However, baseline sex differences in physiology may still be mediating a male liability to autistic traits and eventually autism (11). These sex differences in physiology may be preceding the emergence of autistic traits and emerge in prenatal life and early infancy.

Prenatal testosterone levels, measured in amniotic fluid, have been found to predict autistic traits in infancy (on the Q-CHAT)(12), as well as childhood autistic traits (on the AQ) (13) in the same longitudinal cohort. In addition, estradiol levels in maternal serum (but not testosterone), correlated to the autistic traits of the mother and of the male infants, in a separate study (14). These findings on autistic traits are consistent with reports of elevated prenatal steroids in autistic people, diagnosed with autism in later life (15, 16). However, the evidence remains mixed, particularly when only evaluating testosterone or measuring steroids after birth, in cord blood (17, 18). It is therefore not clear if atypical prenatal steroidogenesis in autism, may also extend to early postnatal life, in the absence of a placenta.

Early infancy is also characterised by a brief increase in steroid hormones, particularly testosterone, at around 2 months of age, a period termed ‘mini-puberty’. This is evident when steroids are measured in blood, urine or saliva (19). Initially this transient increase in androgens was thought to be specific to males and mostly contribute to the development of male genitalia and the differentiation of spermatocytes (20). More recent longitudinal studies show that females also experience high levels of circulating steroids during this period, which further include fluctuating levels of estrogens (21). The latter are produced by the developing ovaries, fluctuate in a way that resembles follicular cycles and eventually decrease in later infancy, albeit in a more gradual way to the surge of circulating testosterone in males (22). Mini-puberty is thought to be induced by the activated HPG axis, following the migration and maturation of GnRh neurons and the absence of placental estrogens, which act to suppress the axis during gestation (23).

The neurodevelopmental significance of mini-puberty remains unclear. Association studies have found links between steroid levels during mini-puberty and language development (7), as well as gender differences in behaviour in childhood, such as play and toy choices (24) However, no association was found in studies of autistic traits, as measured on the Q-CHAT, in two independent studies. The first measured steroids at a later time-point than the predicted peak (at 8 months of age) and controlled for any sex differences in a linear regression model (25). In the second study, the authors were able to measure testosterone in saliva earlier (mean age = 7.8 weeks) but opted for a sex-stratified analysis, potentially reducing power (40 males, 47 females) (26). Neither of these studies included more than one time-point of steroid measurement, or tested for an interaction with infant sex, rather than control or stratify for sex.

To complement these studies, we have investigated the association between mini-puberty testosterone and infant autistic traits in the longitudinal CHILD cohort in Cambridge. CHILD stands for the Cambridge Human Imaging Longitudinal Development study. This included two measurements of salivary hormones, to better capture the peak of mini-puberty, as well as the measuring of autistic traits as an outcome in late infancy (via the Q-CHAT) and the inclusion of a subset of participants from families at high-likelihood for autism (presence of a diagnosed parent or sibling). We hypothesised that increased testosterone levels during mini-puberty would correlate to infant autistic traits, and this association would interact with autism family history and baseline sex differences in testosterone levels.

## Methods

### Participants

Pregnant women were recruited to take part in CHILD at the Rosie Maternity Hospital in Cambridge, *via* advertising material or in-person discussions in the prenatal ultrasound unit, during their first or second pregnancy monitoring appointment. An additional subset of the cohort consisted of pregnant women with a family history of autism, having themselves received a diagnosis of autism, their partner, or a previous child. These “high-likelihood” participants were recruited through the Cambridge Autism Research Database (CARD), support groups across the UK, social media specific to autism, and adverts placed in magazines. Only women with a singleton pregnancy were eligible to take part in the study and those who reported no consumption of alcohol or smoking during pregnancy in the initial screening form for study recruitment. None of the infants included in the study had been born preterm (<37 weeks gestation at birth).

Consent was also obtained for specific linkage to all pregnancy-related clinical data. Birth-related variables, such as birth weight and gestational age at birth, were obtained *via* access on the Cambridge University Hospital (CUH) Trust’s clinical records system (EPIC), by specialist clinicians and anonymised immediately after extraction.

### Salivary hormones

Saliva was collected in the form of passive drool from the infants’ mouth at approximately 2 months of age (mean=10.8[1.9] weeks). This was collected using a validated method from Salimetrics, Ltd., using their Infant Collection Swab (SIS). This consists of an absorbent polymer that absorbs the infant’s saliva, paired with a single-use, single-swab collection tube, in which the swab is placed. Two swabs were used per infant, when possible. Three infants did not tolerate a second swab. Swab-tube pairs were placed in a freezer at 4 degrees Celsius, immediately after collection. Within one hour, these were centrifuged to achieve gradual draining and collection of liquid saliva. They were kept at 4 degrees throughout. The resulting sample was then aliquoted into a cryovial and frozen at -80C. In a few cases (n<4), admixture with tears could not be excluded, given the infant’s distress at the time of sample collection.

The same collection protocol was applied in a second in-person clinical appointment, when the infants were approximately 6 months old (mean=26.5[1.7] weeks). For this subset of samples, collection swabs were immediately frozen at -80C. These were thawed and centrifuged after a period of one year in storage at -80C.

### Hormonal assays for testosterone

An ELISA kit developed and provided by Salimetrics, Ltd (assay #1-2402) was used to measure testosterone concentrations in saliva. This has been validated in samples collected *via* the Infant Collection Swab, as well as in adults, with a reported sensitivity of 1pg/mL, a range between 6.1 and 600pg/mL and a correlation to serum testosterone of 0.96. The detailed protocol, including recommended standards can be found online: https://salimetrics.com/assay-kit/salivary-testosterone-elisa-kit/.

As recommended by Salimetrics, duplicate assays were conducted for each participant’s pooled saliva sample (derived in most cases by two swabs used in quick succession). Final values were the average of the two assays. A quality control step involved conducting a third assay, in case concertation values of the two initial arrays, were not within 15% of each other. The average was then computed, based on the two assays that were closer together. All assays were conducted by specialist staff at the Anglia Ruskin Biomarkers Laboratory, which have been accredited by Salimetrics for this type of analysis.

### Autistic traits

Following informed consent at the point of recruitment, and again before their child’s postnatal appointment for saliva collection, participating mothers were invited *via* email to complete a series of online questionnaires on their child’s development. These were provided *via* the University’s Qualtrics platform. Autistic traits were the first item to be completed, by filling in the Quantitative Checklist of Autism in Toddlers (Q-CHAT) (3). Individualised links to Qualtrics were sent immediately after 18 months had passed, since the child’s birth. Anonymised data was then copied onto RedCap, an online platform for secure storage and data analysis, used by research staff and students of the University of Cambridge (27).

### Statistical analyses

#### Cohort and outcome variables

All variables for the age at the time of postnatal assessments (e.g., age when Q-CHAT was completed) were adjusted for gestational age at birth. For example, an infant being born 2 weeks preterm would result in all postnatal age variables being reduced by 2 weeks. None of the infants were born before 37 weeks gestation. This was done to better reflect the maturation of physiology since conception, rather than birth, as well as control for complications, such as being born preterm, when comparing infants in multiple regression models. For individuals where gestational age at birth was missing from birth records, it was assumed that the neonate had achieved term gestation (40 weeks).

Prior to regression analyses, skewness of the distribution of Q-CHAT scores (the outcome variable) was reduced by reducing extreme outliers (n=2) to a value within an interval of three times the top limit of the interquartile range (Q-CHAT=50.75). Salivary testosterone levels at the 2^nd^ month of life and 6^th^ month of life were log-transformed to reduce skew in their distributions. Comparison between groups (sex or autism likelihood) were conducted with pairwise Student’s t-tests. Associations with demographic variables (e.g., birth weight, age at sampling) were investigated by calculating the Pearson’s correlation coefficient between them and the transformed testosterone concentrations. Three multiple regression models were used to test for an association of testosterone levels during mini-puberty with the autistic traits of the infants at 18 months of age (measured on Q-CHAT); first by controlling for sex (Model 1), second by modelling an interaction with sex (Model 2) and third by adding the second testosterone measurement (Model 3).

## Results

### Hormones at mini-puberty

**Figure 1:**
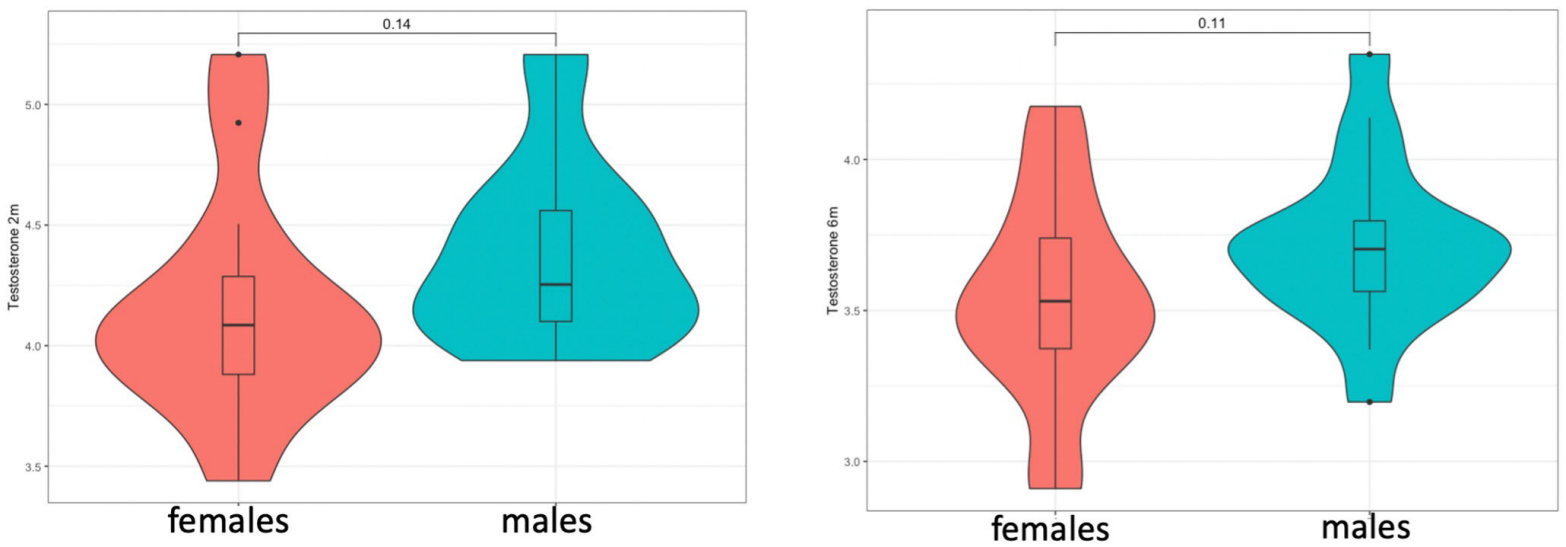
Testosterone levels at 2 (left) and 6 months (right) did not show significant sex differences but showed a consistent trend for higher levels in males.

Testosterone levels in saliva were detected in both males and females in both time-points (1^st^ time-point: n=33, at mean=10.8[1.9] weeks)(2^nd^ time-point: n=34, at mean=26.5[1.7] weeks). These were marginally higher in males at both time-points (**Figure 1**), but this was not statistically significant when these were compared *via* Student’s t-test, potentially due to the low sample size (t-test at 2 months: t=-1.37, p=0.143/t-test at 6 months: t=-1.65, p=0.109). In addition, there were no group differences in testosterone levels between children with a family history of autism and those without, in pairwise comparisons (t-test at 2 months: t=-1.364, p=0.151/t-test at 6 months: t=-1, p=0.327), as well as in linear regression models that controlled for sex. Linear regression models also did not reveal any associations between testosterone and cohort covariates, such as birth weight, maternal age or the exact age at time of saliva collection.

**Figure 2:**
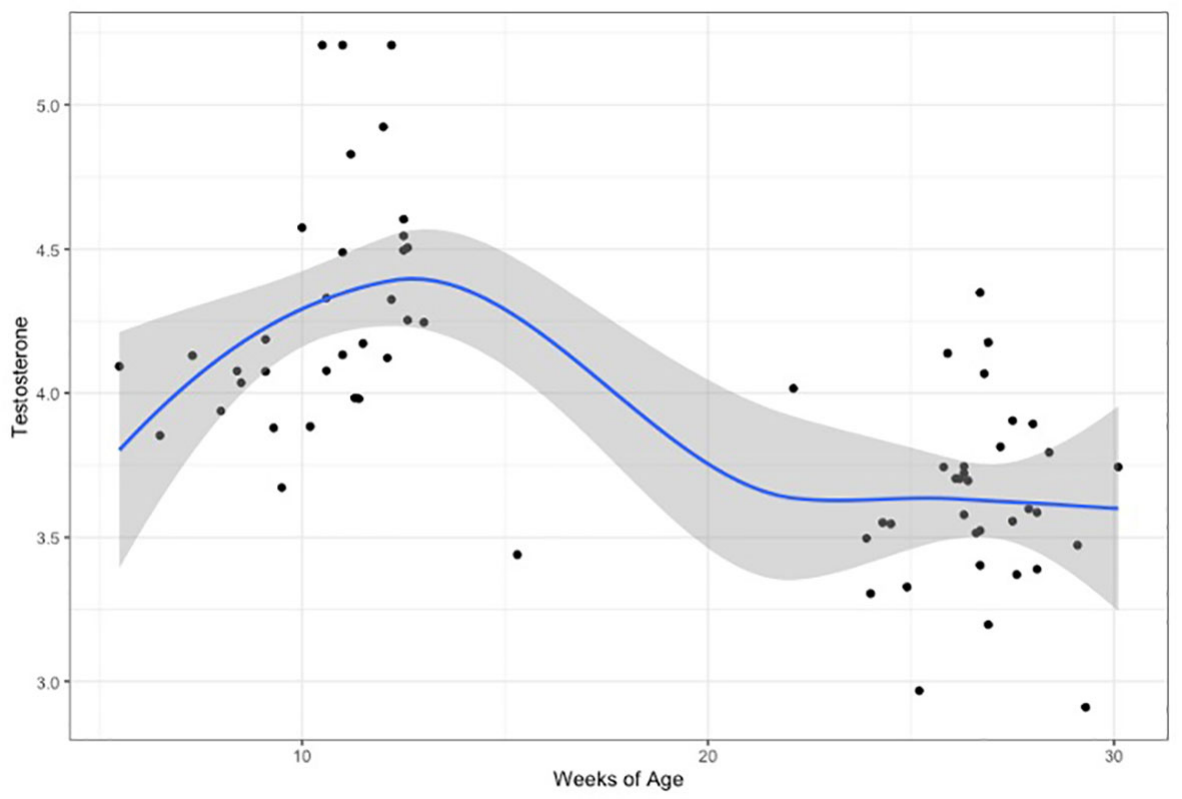
Testosterone levels(log-transformed), plotted for weeks of age (adjusted for gestational age at birth). Curve is loess fit for both time-points.

When comparing hormone levels between time points, testosterone after the 2^nd^ month did not correlate with testosterone levels after the 6^th^ month, but a non-significant trend was noted in females (males: Pearson’s r=0.24, p=0.33/females: Pearson’s r=0.53, p=0.052). Testosterone levels were significantly higher at the first time-point (after 2 months), compared to the second (after 6 months), in both males (t=4.87, p<0.0001) and females (t=3.69, p=0.001). The highest testosterone levels corresponded to 11 to 12 weeks of age (corrected for gestational age at birth) (**Figure 2** and **Supplementary Figure 1**).

### Association with autistic traits

Q-CHAT scores were available for n=36 infants, as rated by their parents (mean=32.4 (SD=10.6)) (**Supplementary Figure 2**). There was no significant difference between males (n=19) and females (n=17) (Student t-test: t=0.91, p=0.372), but infants of families with a history of autism (parent or sibling) scored significantly higher (n=10, mean= 41.6, SD=10.1) than infants with no history of the condition (n=26,mean=28.9, SD=5.7, t-test=2.65, p=0.0242) (**Supplementary Figure 3**). Testosterone levels did not correlate with infant autistic traits in a pairwise Pearson’s correlation coefficient (r=-0.09, p=0.649).

**Figure 3:**
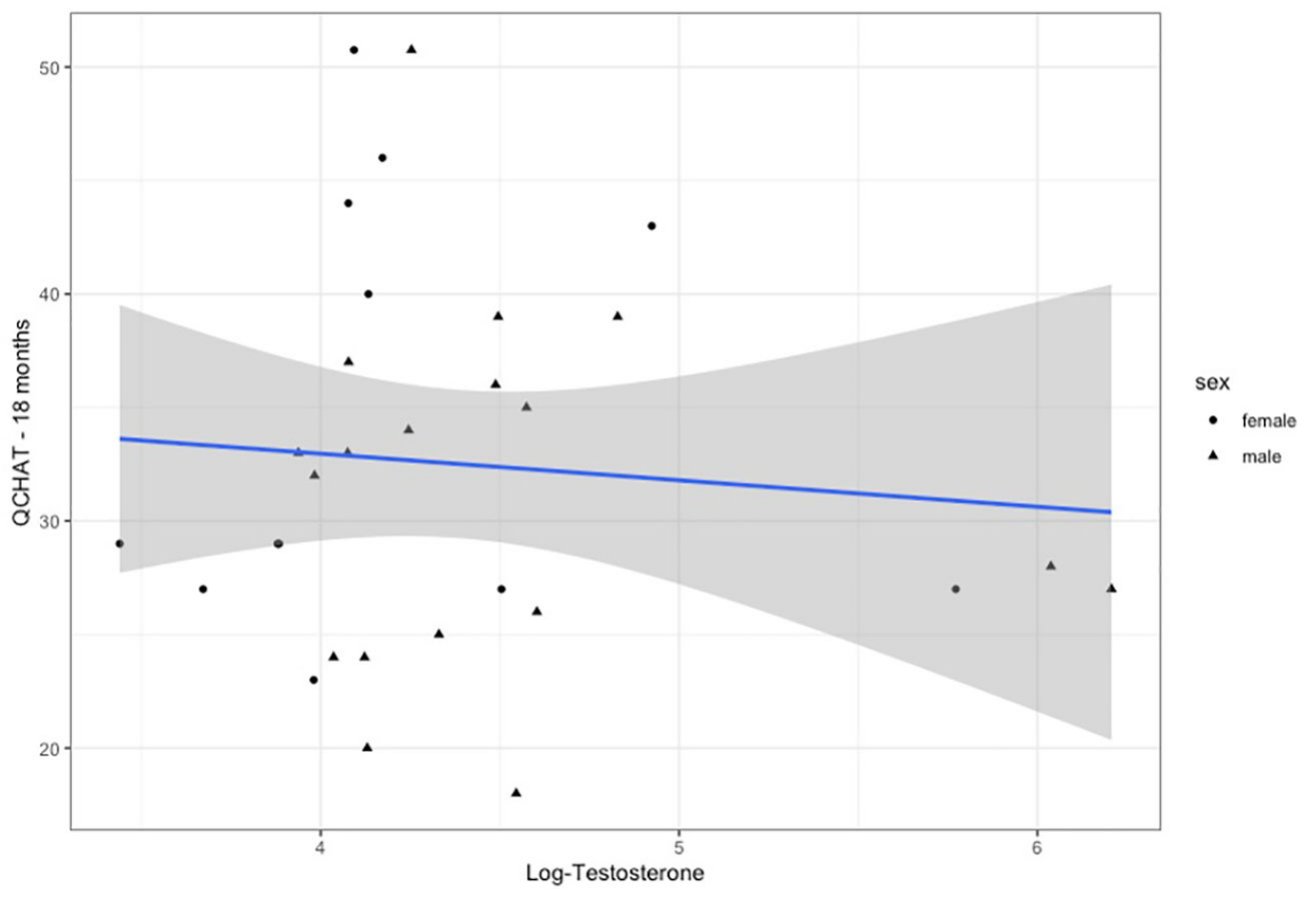
Testosterone levels (log-transformed) plotted for Q-CHAT scores. Linear regression fit is for both males and females.

Three multiple regression models were used to test for an association of testosterone levels during mini-puberty with the autistic traits of the infants at 18 months of age: first by controlling for sex (Model 1), second by modelling an interaction with infant sex (Model 2) and third by adding the second testosterone measurement (Model 3) (see **Table 1**). The age at the time of Q-CHAT and family history of autism were included in all models as covariates. There was no statistical association of testosterone levels with infant autistic traits in any of the models (**Figure 3**). Infant sex or baseline testosterone (at 6 months) also did not have any statistically significant effects in predicting Q-CHAT scores. The only variable that did meet significance was family history of autism, which was significant in all of the models.

**Table 1:** Multiple regression models of log-transformed testosterone levels (T) to autistic traits (Q-CHAT scores).

	*T - 2m*	*age at Q-CHAT*	*sex*	*Autism history*	*T by sex*	*T - 6m*	*T-2m by* *T-6m*
*Model 1*	β=1.14	β=-0.40	β=-1.70	**β=-9.49**			
	p=0.638	p=0.516	p=0.595	**p=0.005**			
	Adj. R^2 =^ 0.191						
*Model 2*	β=1.39	β=-0.39	β=0.104	**β=-9.46**	β=-0.42		
	p=0.721	p=0.533	p=0.996	**p=0.007**	p=0.933		
	Adj. R^2 =^ 0.158						
*Model 3*	β=21.16	β=-0.32	β=-2.86	**β=-9.32**		β=27.19	β=-5.68
	p=0.562	p=0.631	p=0.463	**p=0.010**		p=0.546	p=0.580
						Adj. R^2 =^ 0.115	

“2m”: time-point 2 at months of age, “6m”: time-point at 6 months of age.Bold values indicate statistical significance (p<0.05).

## Discussion

This study did not find any association between testosterone levels during mini-puberty and later autistic traits. This is consistent with previous studies that have investigated the same question (25, 26). Compared to previous attempts, this study was the first to include (1) individuals at high familial likelihood for autism (2) measurement as early as the second month of postnatal life, which better captures the brief spike in steroid levels (3) two consecutive measurements (at mini-puberty and fourth months later at ‘baseline’), in order to better describe this spike and ascertain, for the first time, the extent of this phenomenon in females using salivary testosterone, and finally (4) a statistical model that included an interaction with infant sex, rather than merely controlling for any sex differences that could be mediating both hormone level differences, and differences in autistic traits.

Despite these novel aspects of the study, no link was observed between testosterone levels and autistic traits, as measured by the Q-CHAT questionnaire in early puberty. This is consistent with the null findings in previous studies that measured testosterone in infancy and at birth (in cord blood) (17, 25, 26). In contrast, studies that have examined steroids prenatally, in amniotic fluid, have established links between them and infant autistic traits (12, 28).

This series of findings suggest that *prenatal*, rather than postnatal, brain development is more susceptible to the neurodevelopmental effects of sex steroid hormones. This would place autism-related likelihood at the same developmental window as sex differentiation, a notion that is consistent with the empathising-systemising theory of autism (9, 29). This is because the ‘masculinising’ of both physiology and psychology has been traced back to the ‘prenatal masculinising window’ in the 2^nd^ trimester, a spike in steroid levels in the fetal circulation of males that follows the maturation of the fetal gonads (30, 31).

Nevertheless, organisational effects of postnatal testosterone cannot be excluded and indeed have been proposed to lead to more ‘masculine’ play behaviour in childhood in recent studies (32). In addition, perinatal factors such as preterm birth, mode of delivery and the metabolic health of the mother may be confounding a relationship between mini-puberty testosterone and autistic traits (33, 34). Non-linear associations may also be relevant, as low steroid levels could also be reflecting endocrine dysfunction coupled with atypical neurodevelopment, as in the case of hypogonadism and autistic traits in Klinefelter’s syndrome (35).

The relative significance of prenatal, rather than postnatal surges in sex steroids in autism, would implicate steroid synthesis pathways that are specific to pregnancy, such as those involving the placenta. The placenta metabolises androgens rapidly to estrogens and also synthesises a variety of other steroids and neurotransmitter precursors throughout pregnancy. A link between placental steroid synthesis is further indicated by recent findings on associations of prenatal estrogens in humans and placenta-derived allopregnanolone in animals, with autistic traits (14, 36). Interestingly, in both organisms, the association significantly interacted with the sex of the offspring, with prenatal, placenta-related steroids affecting males and females differently. This could be related to baseline sex differences in placental functionality, which include both difference in steroid hormone levels, but also molecular differences in terms of X-chromosome inactivation and gene expression levels (37, 38).

Finally, the factors regulating placental steroidogenesis and mini-puberty may not overlap, since the fetal HPG axis and GnRh neurons are not yet fully independent during prenatal life, but rather form only one part of the maternal-placental-fetal unit. This is further indicated by a lack of correlation between fetal and infant levels of testosterone, as shown in longitudinal studies of the same individuals (25). In addition, anatomical features, such as AGD and penile length, differ in their association to androgen timings, reflecting steroid levels in early gestation and mini-puberty respectively (39, 40), but not vice versa (41). Interestingly, AGD and penile length also appear to be independent in their association to later gendered behaviour and play (42, 43). Therefore, additional research is needed to study the similarities and the differences in the regulation of prenatal and early postnatal steroids, as well as their link to various aspects of neurodevelopment.

This study confirms that salivary testosterone is elevated in females, during mini-puberty, albeit to a lesser extent than the one in males. Previous longitudinal studies of the phenomenon in females have relied on urinary secretion of the hormone (32). Only one study assessed the venous circulation of infants directly (n=32) and reported a gradual decrease of testosterone in females since birth, rather than a transient elevation in the second and third month of life, as seen in males. They reported much higher testosterone levels in males than females in the second month of life (more than a factor of 8x difference) (44). However, this study found considerable overlap in salivary testosterone levels between the sexes and no statically significant differences between males and females, at either 2 months or 6 months of age. This is consistent with previous studies using salivary measures of testosterone, which also failed to find a statistically significant sex difference during and after mini-puberty, despite having larger sample size (25, 45). Saliva may then not be the most appropriate sample for the study of sex differences, particularly since it corresponds to the unbound fraction of steroids in the circulation.

This study is limited considerably by its low sample size, compared to previously successful studies of mini-puberty. For example, with a sample size of n=77, researchers were able to uncover a correlation of steroid levels during this period, with the size of expressive vocabulary (46). Smaller cohort studies (n<30) have reported language-related associations, but only when they included physically measured outcomes, rather than parent-reported psychometric measures (e.g. phonetic articulation analyses of voice recordings or brain imaging *via* EEG) (7, 47). The developmental window of mini-puberty may then better correspond to associated cognitive traits of autism, such as language development, rather than core features such as social inattention and repetitive behaviours.

In conclusion, this study reports higher levels of mini-puberty testosterone in the saliva of both male and female infants, but finds no association with their later autistic traits, suggesting that prenatal, rather than postnatal steroidogenesis may be more relevant for understanding the causes of autism.

## Data availability statement

The datasets presented in this article are not readily available because access to the raw data pertaining to the participants of this study is restricted to members of the research team which received approval and consent to analyse them. Requests to access the datasets should be directed to at768@medschl.cam.ac.uk.

## Ethics statement

The studies involving human participants were reviewed and approved by East of England - Cambridgeshire and Hertfordshire Research Ethics Committee (Reference: 12/EE/0393). Written informed consent to participate in this study was provided by the participants’ legal guardian/next of kin.

## Author contributions

AT conducted the analysis, interpreted the data and drafted the manuscript. SH assisted with sample retrieval and storage. EA assisted with the setup of the study and the recruitment of the study participants. CA advised on the assessment of infant neurodevelopment and use of questionnaires. SBC and RH contributed equally to study design, study supervision, data interpretation and to the revisions of the manuscript. All authors contributed to the article and approved the submitted version.
