# Supplementary material for: Mini-puberty testosterone and infant autistic traits
Source: Front Endocrinol (Lausanne). 2023 Apr 5;14:1126023. doi: 10.3389/fendo.2023.1126023 (PMC10113441; doi:10.3389/fendo.2023.1126023)
Supplement: Supplementary file 1 [file DataSheet_1.pdf]

Supplementary Material

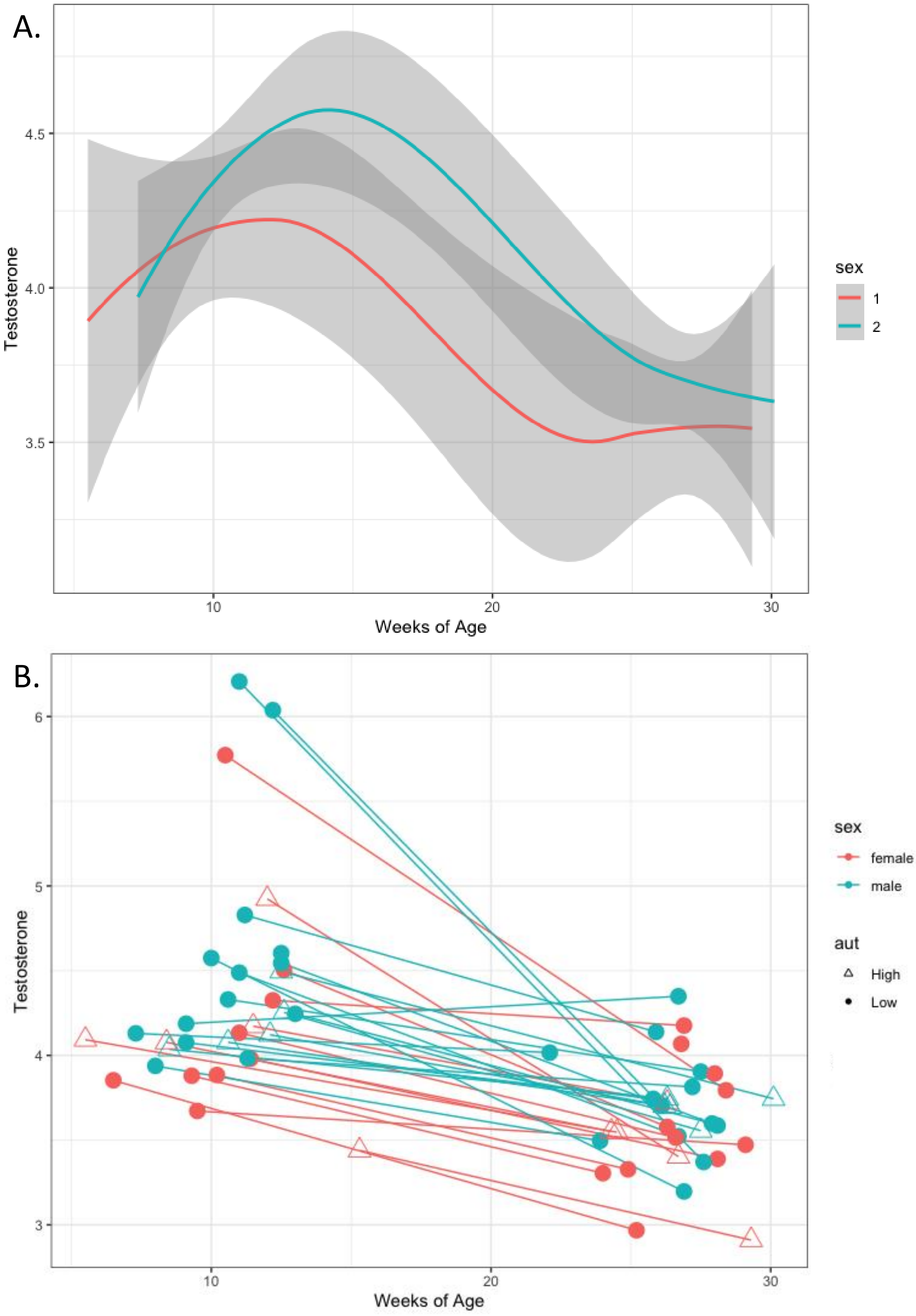

Suppl. Figure 1: Testosterone levels for age since birth (in weeks). (A) Loess curves for each sex indicate a mini-puberty elevation for both males and females (B) Paired concertation values for each participant, for sex (1: female, 2: male) and familial likelihood.

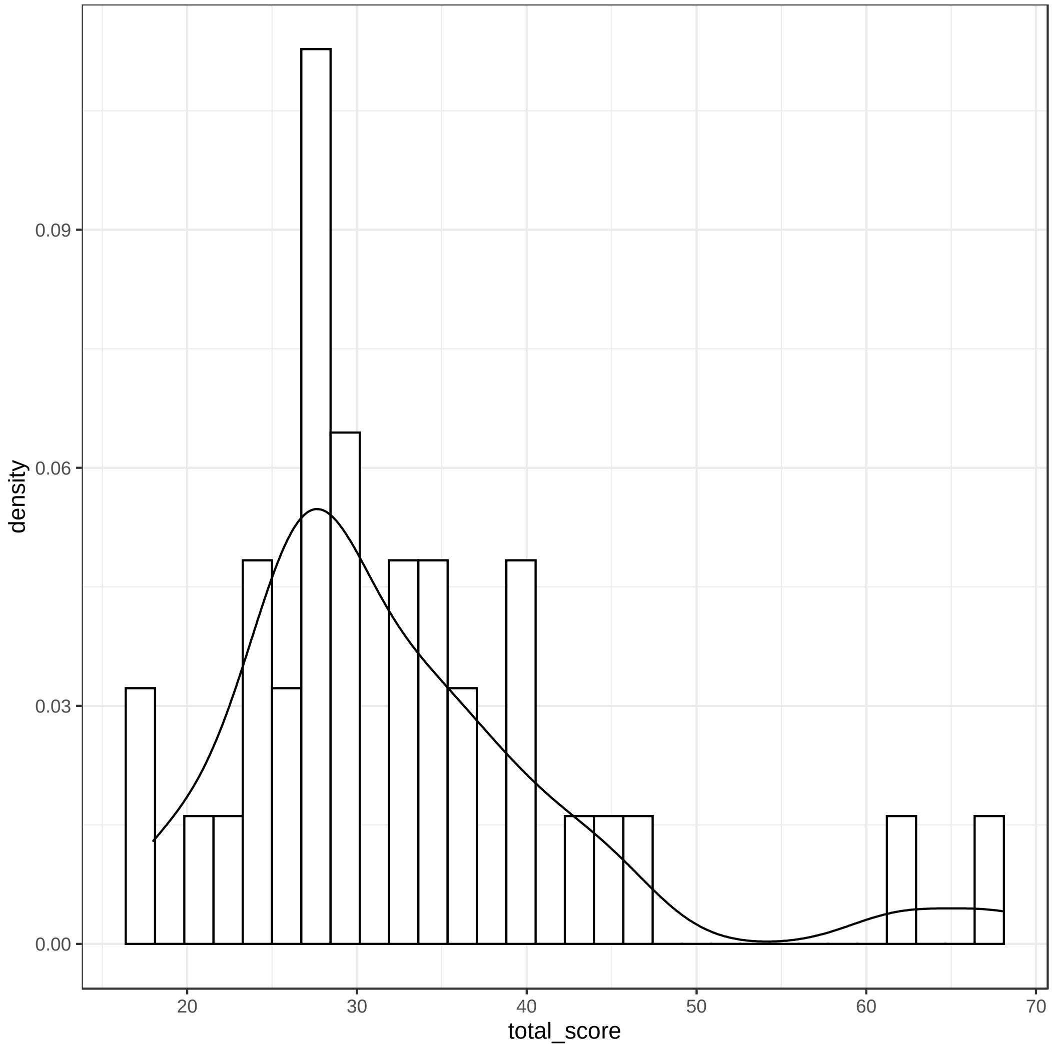

Suppl. Figure 2: Histogram and density line of Q-CHAT scores at 18 months in the CHILD cohort. High ouliers werer windorised prior to statistica analysis.

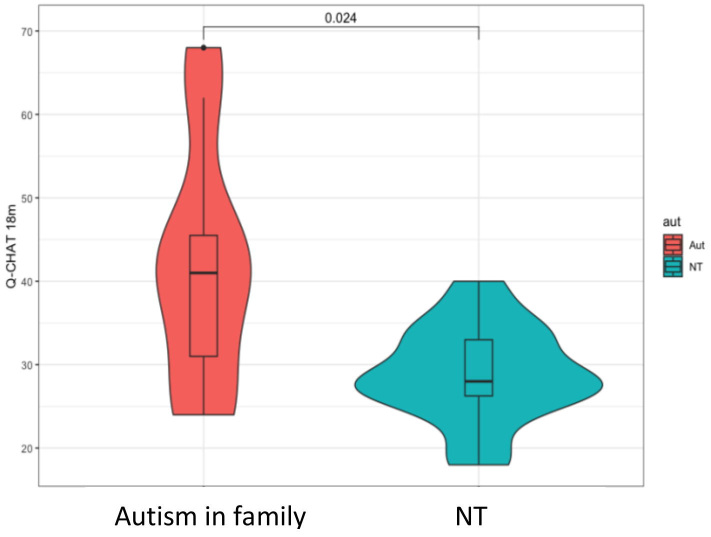

Suppl. Figure 3: Boxplot of Q-CHAT scores at 18 months, showing a significant difference between infants at high familial likelihood of autism, based on a diagnosed parent or sibling ('Autism in family') and those without ('NT').
Bar shows p-value of Student's t-test.
